# Comprehensive analysis reveals the involvement of hsa_circ_0037858/miR-5000-*3p/FMR1* axis in malignant metastasis of clear cell renal cell carcinoma

**DOI:** 10.18632/aging.204790

**Published:** 2023-06-27

**Authors:** Yuan Nianyong, Guowei Li

**Affiliations:** 1 Department of Hepatobiliary and Pancreatic Surgery, The First People’s Hospital of Fuyang, Hangzhou 311400, China

**Keywords:** circular RNA (circRNA), microRNA (miRNA), clear cell renal cell carcinoma (ccRCC), metastasis

## Abstract

Clear cell renal cell carcinoma (ccRCC) is a heterogenous tumor with high metastatic potential. Circular RNAs (circRNAs) play key roles in cancer initiation and progression. However, the knowledge of circRNA in ccRCC metastasis is still inadequate. In this study, a series of *in silico* analyses and experimental validation were employed. The differentially expressed circRNAs (DECs) between ccRCC and normal or metastatic ccRCC tissues were screened out using GEO2R. Hsa_circ_0037858 was identified as the most potential circRNA related to ccRCC metastasis, which was significantly downregulated in ccRCC compared with normal and was also markedly decreased in metastatic ccRCC compared with primary ccRCC. The structural pattern of hsa_circ_0037858 presented several microRNA response elements and four binding miRNAs of hsa_circ_0037858, consisting of miR-3064-5p, miR-6504-5p, miR-345-5p and miR-5000-3p, were predicted using CSCD and starBase. Among them, miR-5000-3p with high expression and statistical diagnostic value was considered as the most potential binding miRNA of hsa_circ_0037858. Then, protein-protein interaction analysis revealed a close linkage among the target genes of miR-5000-3p and the top 20 hub genes among them were identified. Based on node degree, *MYC*, *RHOA*, *NCL*, *FMR1* and *AGO1* were ranked as the top 5 hub genes. *FMR1* was identified as the most potential downstream gene of hsa_circ_0037858/miR-5000-3p axis according to expression, prognosis and correlation analysis. Moreover, hsa_circ_0037858 suppressed *in vitro* metastasis and enhanced *FMR1* expression in ccRCC, which could be markedly reversed by introduction of miR-5000-3p overexpression. Collectively, we elucidated a potential hsa_circ_0037858/miR-5000-3p/*FMR1* axis involved in ccRCC metastasis.

## INTRODUCTION

Renal cell carcinoma (RCC), originating from the renal epithelial cells, is a common malignant solid tumor of urinary system, accounting for 80-90% of all kidney malignancies [1, 2]. RCC tumors are extremely heterogenous with multiple histological subtypes, among which clear cell renal cell carcinoma (ccRCC) is the most frequent one, making up nearly 70% of all kidney malignancies [3]. With the ability to invade renal sinus and extend to the renal vein, ccRCC is characterized by high metastatic potential, which causes approximately 30% of ccRCC patients possessing metastatic disorders at the first time of diagnosis [4]. Therefore, it makes sense to uncover the molecular mechanism of ccRCC metastasis.

Circular RNAs (circRNAs) are a class of novel, endogenous and noncoding RNAs with the feature of covalently closed loops [5, 6]. CircRNAs are more stable than their linear counterparts as they have no 5’-cap and 3’-polyadenylated tail structure [7, 8]. During the past years, increasing lines of evidence have found that circRNAs play key roles in initiation and progression of multiple human malignancies, including breast cancer [9, 10], papillary thyroid carcinoma [11, 12], hepatocellular carcinoma [13, 14] and colorectal cancer [15, 16]. Intriguingly, several circRNAs have also been reported to participate in biological process of ccRCC metastasis. For example, circ-TNPO3 hindered ccRCC metastasis by binding to IGF2BP2 [17]; circular RNA ITCH suppressed ccRCC metastasis by regulating miR-106b-5p/PDCD4 axis [18]; circ-AKT3 inhibited ccRCC metastasis through changing miR-296-3p/E-cadherin signals [19]. However, to date, our knowledge of circRNAs in metastasis of ccRCC is still inadequate and needs to be further studied.

In this work, we firstly screened out and validated candidate ccRCC metastasis-related circRNAs by analyzing two GEO datasets (GSE100186 and GSE137836). Then, the structural pattern of potential circRNA and its parental gene were obtained. Subsequently, the binding miRNAs of potential circRNA and the downstream target genes of potential circRNA/miRNA axis were predicted and analyzed by using a series of online databases or tools. At the end, a potential circRNA/miRNA/mRNA triple regulatory axis involved in the process of ccRCC metastasis was successfully constructed.

## MATERIALS AND METHODS

### Inclusion of datasets

In this study, to explore the role and mechanism of circRNA in malignant metastasis of ccRCC, the potential datasets from the NCBI GEO database (http://www.ncbi.nlm.nih.gov/geo/) were included. The included datasets should meet the following criteria: (1) the included datasets should study the circRNA expression profile in ccRCC and/or normal tissues; (2) the datasets regarding cell lines should be excluded; (3) the datasets focusing on the circRNA expression in metastasis of ccRCC should also be included. At the end, only two datasets, consisting of GSE100186 and GSE137836, met the above selection criteria. Both GSE100186 and GSE137836 were based on the platform of GPL21825 074301 Arraystar Human CircRNA microarray V2. GSE100186 contained 4 ccRCC and 4 matched non-tumor samples and GSE137836 contained 3 primary tumor tissues and 3 ccRCC metastatic tumor tissues from 3 ccRCC patients.

### Differential expression analysis

As previously described [11], the online tool GEO2R (http://www.ncbi.nlm.nih.gov/geo/geo2r/) from the NCBI GEO database (http://www.ncbi.nlm.nih.gov/geo/) was employed to obtain differentially expressed circRNAs between ccRCC and normal tissues or metastatic ccRCC by performing differential expression after conducting sample data normalization.

### Intersection analysis

VENNY 2.1 (http://bioinfogp.cnb.csic.es/tools/venny) tool was introduced to conduct intersection analysis to acquire the potential circRNAs that were commonly appeared in GSE100186 and GSE137836. Moreover, this tool was also used to obtain the common binding miRNAs of hsa_circ_0037858 from CSCD and starBase databases.

### Expression validation for circRNAs

The expression levels of three potential circRNAs related to malignant metastasis of ccRCC were further validated by extracting their expression data from GSE100186 and GSE137836, after which *t*-test was employed to calculate the expression difference of them in normal, ccRCC and metastatic ccRCC tissues.

### circBase analysis

CircBase (http://www.circbase.org/) [20], a database providing scripts to obtain known and novel circRNAs in sequencing data, was introduced to acquire the genome location and parental gene of hsa_circ_0037858.

### Cancer-specific CircRNA database (CSCD) analysis

CSCD (https://gb.whu.edu.cn/CSCD) [21], a cancer-specific circRNA database for predicting the potential MREs, RBPs and open reading frame of circRNAs, was utilized to generate the structural pattern and forecast the binding miRNAs of hsa_circ_0037858.

### miRNA prediction

In addition to CSCD as mentioned above, another database, namely starBase (http://starbase.sysu.edu.cn/) [22], was also employed to predict the possible miRNAs that could potentially bind to hsa_circ_0037858. Only the binding miRNAs that were commonly appeared in both CSCD and starBase databases were included for subsequent analysis.

### Expression determination for miRNAs

The expression levels of binding miRNAs of hsa_circ_0037858 in ccRCC were determined using starBase (http://starbase.sysu.edu.cn/) [22]. Only P-value < 0.05 was considered as statistically significant.

### CancerMIRNome analysis

CancerMIRNome (http://bioinfo.jialab-ucr.org/CancerMIRNome) [23] is an integrated analysis and visualization database for miRNome profiles of human cancer, which was introduced to validate the expression of 4 potential binding miRNAs of hsa_circ_0037858 in ccRCC. Furthermore, the database was also used to assess the diagnostic values of them in ccRCC.

### Target gene prediction

The possible target genes of miR-5000-3p were predicted by usage of a series of online target gene prediction tools, including PITA, RNA22, miRmap, microT, miRanda, PicTar and TargetScan. Only the predicted target genes that were commonly appeared in more than 2 tools were included for subsequent analysis.

### STRING analysis

STRING database (https://string-db.org/) is an online tool aiming to integrate all known and predicted associations between proteins, containing physical interactions and functional associations [24]. The database was used to construct a protein-protein interaction (PPI) regulatory network of target genes of miR-5000-3p.

### Identification of hub genes

The hub genes among the target genes of miR-5000-3p were identified with the help of STRING database and Cytoscape software. At the beginning, the interaction gene pairs among the target genes were downloaded from STRING database. Then, these gene pairs were re-entered into Cytoscape software. Finally, the node degree of individual gene was calculated by Cytohubba. According to the node degree, the top 20 hub genes were screened out.

### Kaplan-Meier plotter analysis

Kaplan-Meier plotter database (http://kmplot.com/) is capable to access the relationship between the expression levels of all genes (mRNA, miRNA, protein) and survival in more than 20 tumor types, including ccRCC [25]. In this study, this database was utilized to evaluate the prognostic values of the top 20 hub genes in ccRCC.

### Correlation analysis

Expression correlation between miRNAs and target genes could provide key clues to identify potential miRNA-target gene pairs based on the negative role of miRNAs in regulating corresponding target genes. Thus, correlation analysis was employed for determining the expression relationship between miR-5000-3p and the top 20 hub genes in ccRCC using starBase database (http://starbase.sysu.edu.cn/) as previously described [26].

### Cell lines, cell culture and cell transfection

Human ccRCC cell lines ACHN and Caki-1 were purchased from the Chinese Academy of Sciences (Shanghai, China) and were cultured in DMEM supplemented with 10% FBS under a humidified atmosphere of 5% CO_2_ at 37° C. circRNA overexpressed plasmid, miRNA mimic and corresponding negative controls were transfected into ccRCC cells by usage of Lipofectamine™ 3000 based on the manufacturer’s instruction.

### RNA isolation and qRT-PCR analysis

RNAiso plus Reagent was used to extract the total RNAs from cell lines, after which RNAs were reversely-transcribed into cDNA. Next, PCR was performed in triplicates using a Roche LightCycler480 II Real-Time PCR Detection System by SYBR Premix Ex Taq (TaKaRa, RR420A). Finally, FMR1 expression (relative to GAPDH) was calculated by the method of 2^-ddCt^.

### *In vitro* migration assay

The *in vitro* migration assay was conducted by 24-well transwell chambers (Corning, USA). The transfected cells were re-cultured with serum-free medium for 24 hours, after which cells were suspended in 0.2 ml serum-free medium and added into the upper chamber and medium containing 10% FBS was added into the lower chamber following 24-hour culture. Subsequently, the cells on the lower surface of the membrane were fixed and stained by 100% methanol and 0.1% crystal violet, respectively. Finally, the cells were counted under a microscope.

### Statistical analysis

Most of the statistical analyses in this study were automatically calculated by the online databases and tools as mentioned above. Expression determination between two groups was analyzed by GraphPad Prism software (Version 7). P-value < 0.05 or logrank P-value < 0.05 was considered as statistically significant.

### Availability of data and materials

All data and materials have been provided in the manuscript. Please do not hesitate to contact the corresponding author.

## RESULTS

### Identification of hsa_circ_0037858 as a potential circRNA related to metastasis of ccRCC

**Figure 1:**
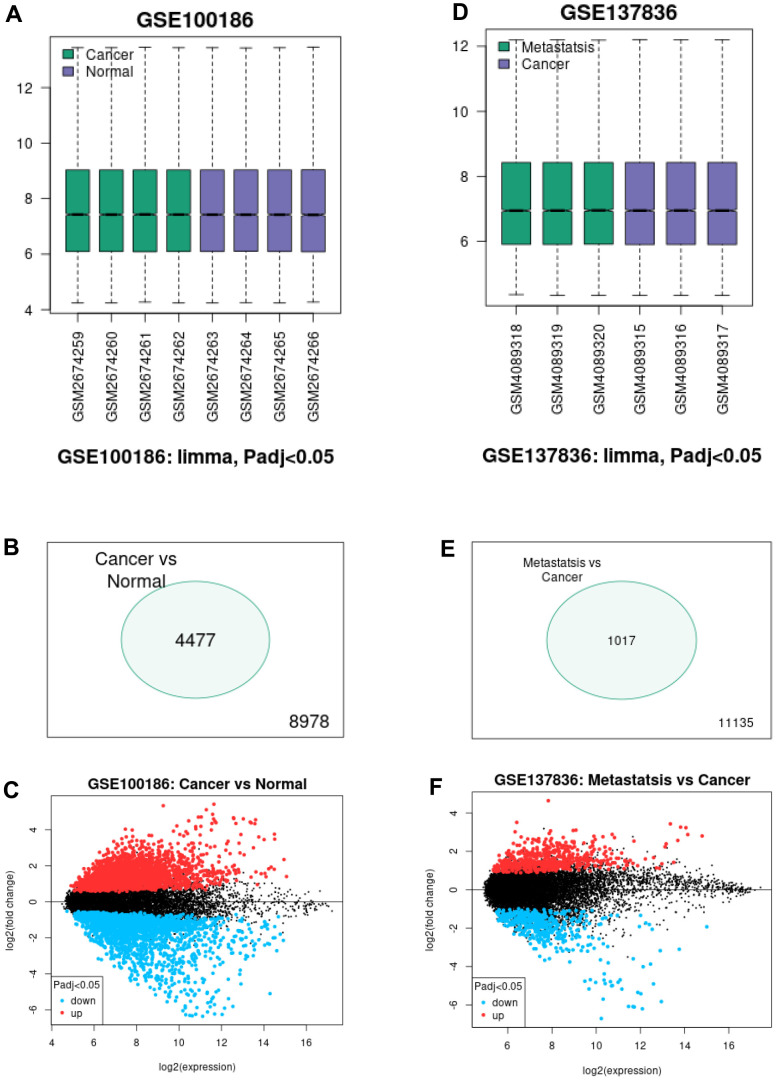
**Screening of differentially expressed circRNAs (DECs) associated with malignant metastasis of ccRCC.** (**A**) The normalization of samples in GSE100186 analyzed by GEO2R online tool. (**B**) The significant DECs between ccRCC cancer tissues and normal tissues. (**C**) The volcano plot of the DECs between ccRCC cancer tissues and normal tissues in GSE100186. (**D**) The normalization of samples in GSE137836 analyzed by GEO2R online tool. (**E**) The significant DECs between ccRCC cancer tissues and metastatic cancer tissues. (**F**) The volcano plot of the DECs between ccRCC cancer tissues and metastatic cancer tissues in GSE137836.

**Figure 2:**
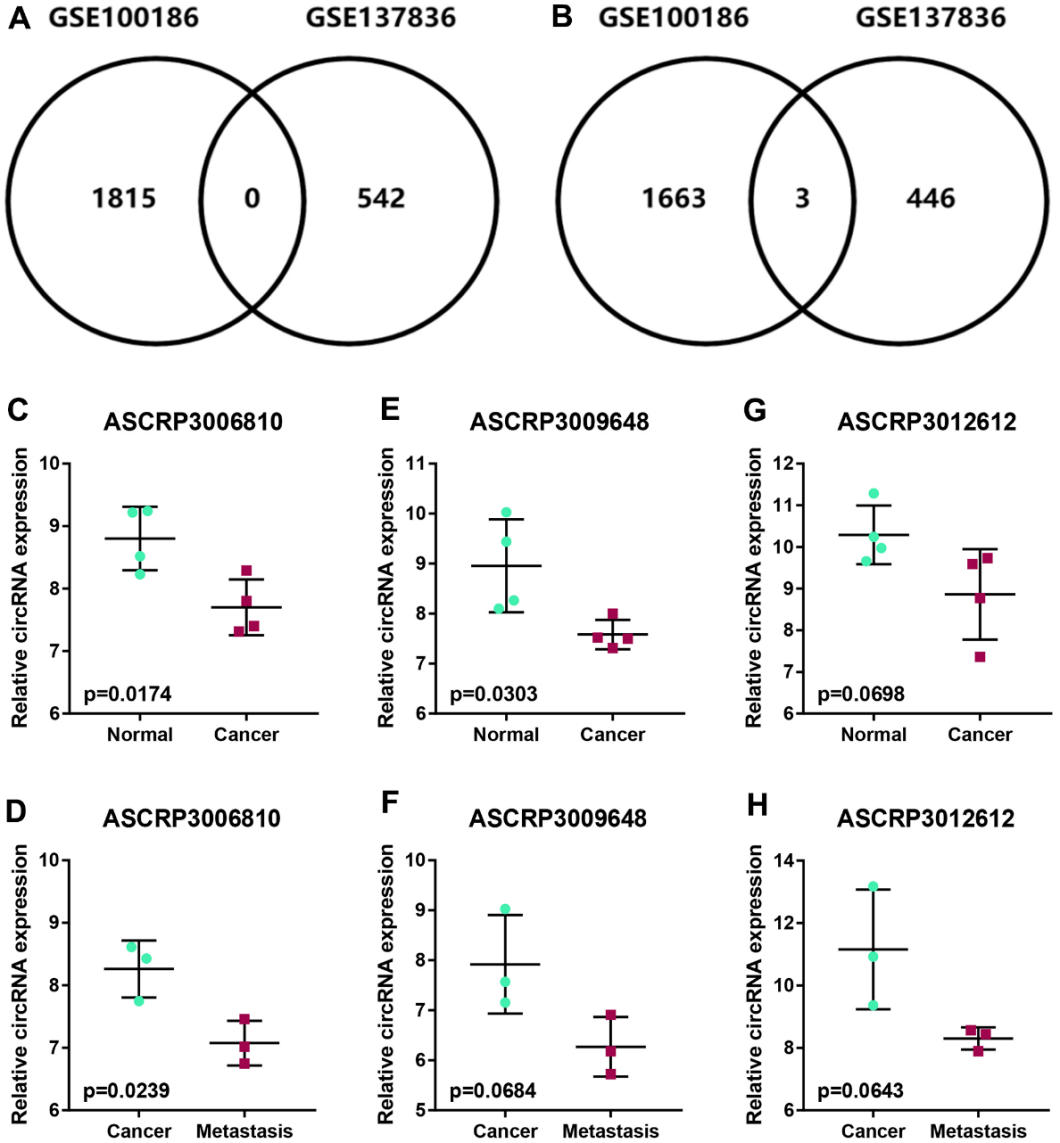
**Identification of potential circRNAs associated with metastasis of ccRCC.** (**A**) The intersection analysis for the significant upregulated circRNAs with |log_2_FC| > 1 in GSE100186 and GSE137836. (**B**) The intersection analysis for the significant downregulated circRNAs with |log_2_FC| > 1 in GSE100186 and GSE137836. (**C**, **D**) The expression levels of ASCRP3006810 in normal, cancer and metastatic cancer tissues (ccRCC). (**E**, **F**) The expression levels of ASCRP3009648 in normal, cancer and metastatic cancer tissues (ccRCC). (**G**, **H**) The expression levels of ASCRP3012612 in normal, cancer and metastatic cancer tissues (ccRCC).

To intend to explore the role and mechanism of circRNA in malignant metastasis of ccRCC, two GEO datasets, consisting of GSE100186 and GSE137836, were included in this study. By using GEO2R tool, data normalization and differential expression analysis for GSE100186 and GSE137836 were performed (Figure 1). A total of 4477 significant differentially expressed circRNAs (DECs) were identified in ccRCC compared with normal controls in GSE100186, consisting of 2281 upregulated and 2196 downregulated DECs (Figure 1B). 1017 DECs were significantly differentially expressed between ccRCC and metastatic ccRCC tissues in GSE137836, containing 567 upregulated and 450 downregulated DECs (Figure 1C). Next, intersection analysis for the two datasets was performed (Figure 2A, 2B). The result suggested that three downregulated circRNAs were commonly appeared in both GSE100186 and GSE137836, including ASCRP3006810, ASCRP3009648 and ASCRP3012612. To improve the analytic accuracy, the expression data of the three potential circRNAs from GSE100186 and GSE137836 were downloaded and re-analyzed (Figure 2C–2G). The results showed that only ASCRP3006810 (hsa_circ_0037858) was markedly downregulated in ccRCC compared with normal controls (Figure 2C) as well as decreased in metastatic ccRCC compared with primary cancer tissues (Figure 2D). All these findings suggested that hsa_circ_0037858 might be a potential tumor suppressive circRNA in metastasis of ccRCC.

### The structural characteristics of hsa_circ_0037858

**Figure 3:**
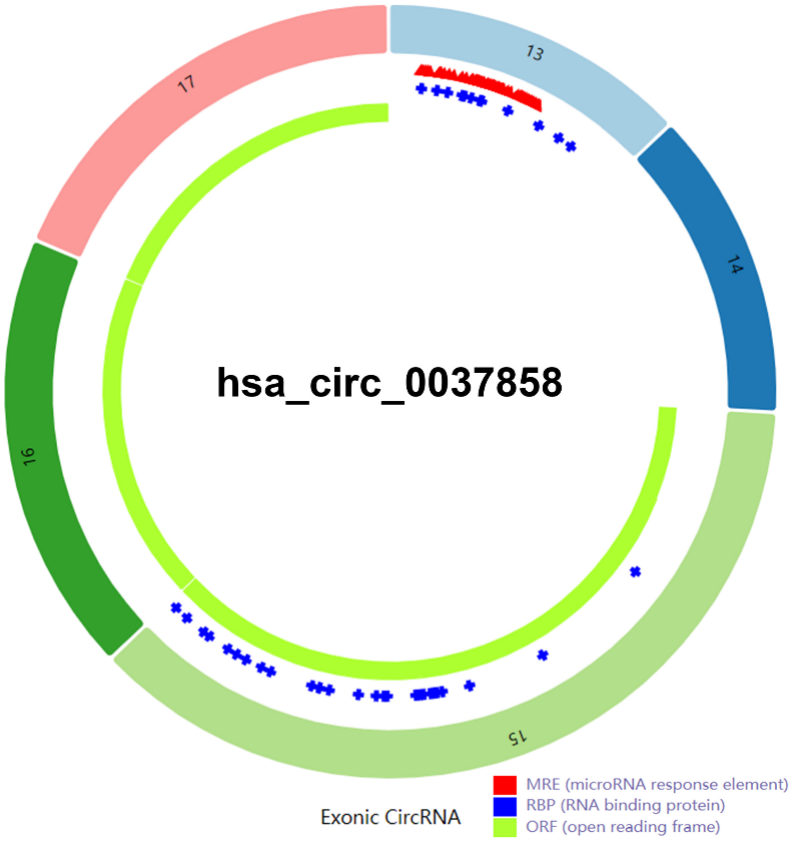
The structural pattern of exonic hsa_circ_0037858, consisting of exon 13, 14, 15, 16 and 17.

To further understand hsa_circ_0037858, circBase database was employed. The genome position of hsa_circ_0037858 was chr16: 11647388-11650591, and the parental gene of hsa_circ_0037858 was LITAF. Next, Cancer-Specific CircRNA Database (CSCD) database was utilized to generate the structural pattern of hsa_circ_0037858. As shown in Figure 3, hsa_circ_0037858 was generated by exons 13, 14, 15, 16 and 17. Intriguingly, hsa_circ_0037858 possessed several microRNA response elements, RNA binding protein binding domain and open reading frame, representing three potential action mechanisms of circRNA.

### Identification of 4 potential binding miRNAs of hsa_circ_0037858

**Figure 4:**
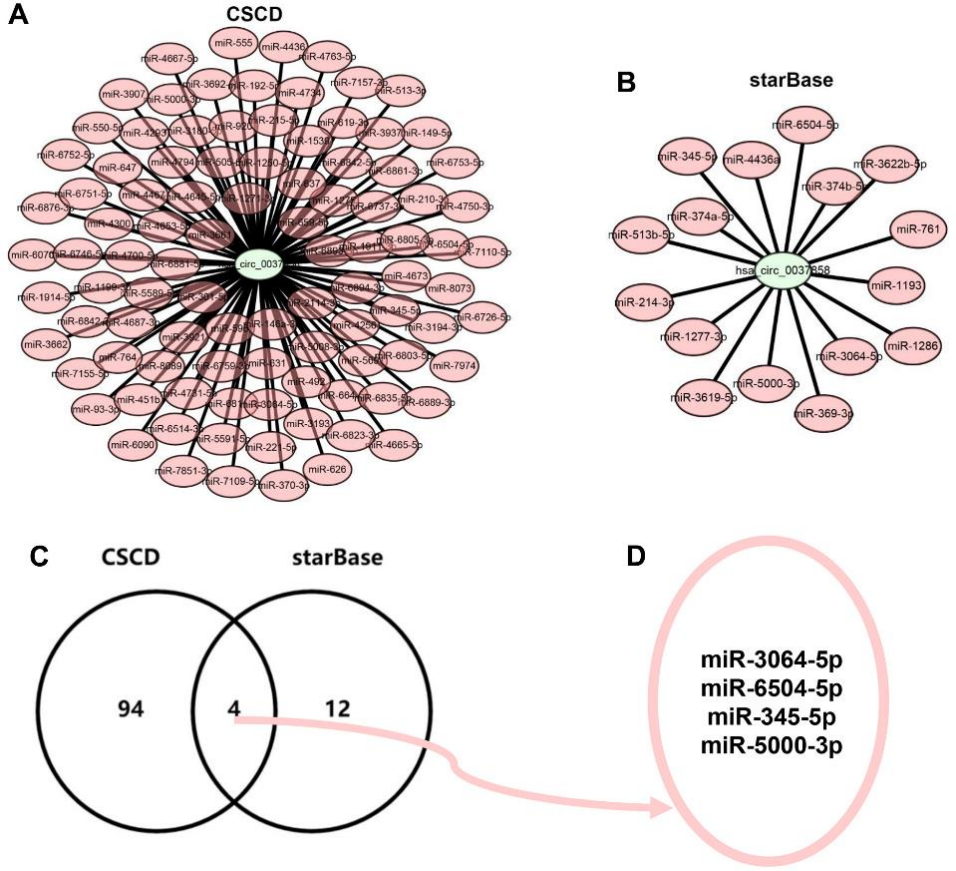
**Identification of potential binding miRNAs of hsa_circ_0037858.** (**A**) The binding miRNAs of hsa_circ_0037858 predicted by CSCD database. (**B**) The binding miRNAs of hsa_circ_0037858 predicted by starBase database. (**C**) The intersection analysis for the binding miRNAs predicted by CSCD and starBase databases. (**D**) miR-3064-5p, miR-6504-5p, miR-345-5p and miR-5000-3p are commonly appeared in CSCD and starBase databases.

It has been widely acknowledged that miRNA binding is the main classic action mechanism of circRNA. Two databases, consisting of CSCD and starBase, were introduced to predict the binding miRNAs of hsa_circ_0037858. A total of 98 and 16 miRNAs of hsa_circ_0037858 were forecasted in CSCD and starBase, respectively. To better visualization, hsa_circ_0037858-miRNA regulatory networks were constructed as presented in Figure 4A, 4B. Subsequently, intersection analysis for miRNAs of hsa_circ_0037858 from CSCD and starBase databases were performed (Figure 4C). The results indicated that 4 miRNAs, consisting of miR-3064-5p, miR-6504-5p, miR-345-5p and miR-5000-3p, were commonly appeared in CSCD and starBase databases (Figure 4D). Taken together, miR-3064-5p, miR-6504-5p, miR-345-5p and miR-5000-3p might be the most potential binding miRNAs of hsa_circ_0037858.

### Analysis for the 4 potential binding miRNAs of hsa_circ_0037858 in ccRCC

**Figure 5:**
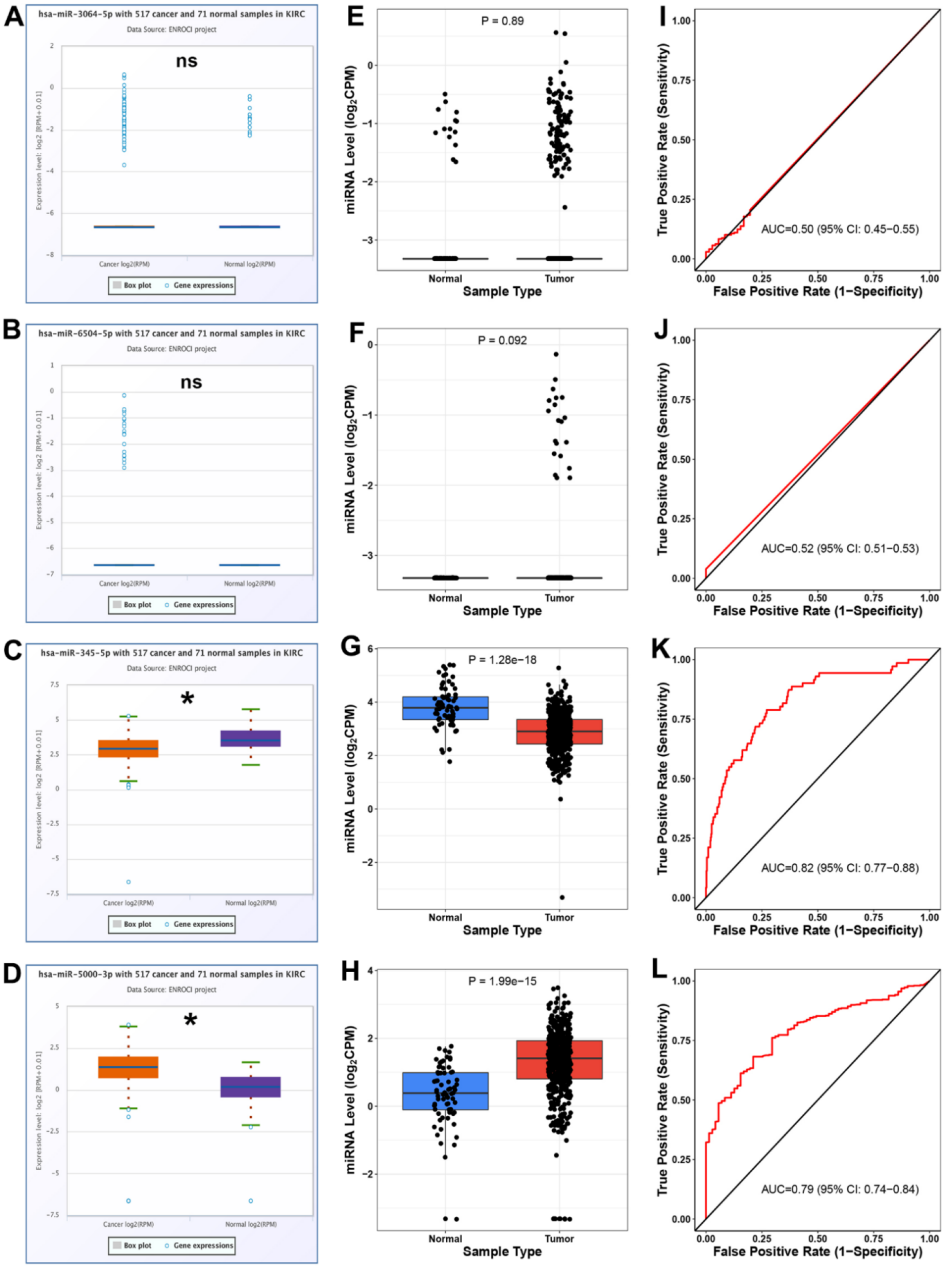
**Analysis for the expression and diagnostic values of 4 potential miRNAs of hsa_circ_0037858 in ccRCC.** The expression levels of miR-3064-5p (**A**), miR-6504-5p (**B**), miR-345-5p (**C**) and miR-5000-3p (**D**) in ccRCC compared with normal controls analyzed by starBase. The expression levels of miR-3064-5p (**E**), miR-6504-5p (**F**), miR-345-5p (**G**) and miR-5000-3p (**H**) in ccRCC compared with normal controls analyzed by CancerMIRNome. The ROC curves of miR-3064-5p (**I**), miR-6504-5p (**J**), miR-345-5p (**K**) and miR-5000-3p (**L**) in ccRCC analyzed by CancerMIRNome. nsP>0.05; *P<0.05.

Expression analysis for the four potential binding miRNAs of hsa_circ_0037858 in ccRCC was conducted using starBase database. As shown in Figure 5A–5D, among the 4 miRNAs, miR-345-5p was significantly downregulated and miR-5000-3p were markedly upregulated in ccRCC when compared with normal controls. Next, CancerMIRNome database was used to validate the expression levels of miR-3064-5p, miR-6504-5p, miR-345-5p and miR-5000-3p in ccRCC (Figure 5E–5H). Identical with the analytic result from starBase, miR-345-5p and miR-5000-3p were statistically downregulated and upregulated in ccRCC when compared with normal controls, respectively. Subsequently, ROC curve analysis by CancerMIRNome database was employed to assess the diagnostic values of miR-3064-5p, miR-6504-5p, miR-345-5p and miR-5000-3p in ccRCC (Figure 5I–5L). The results showed that only 2 of 4 miRNAs (miR-345-5p and miR-5000-3p) possessed the obvious abilities to distinguish ccRCC tissues from normal tissues. All these findings suggested that miR-5000-3p might be the most potential binding miRNA of downregulated hsa_circ_0037858 in ccRCC.

### Protein-protein interaction (PPI) analysis for the target genes of miR-5000-3p

**Figure 6:**
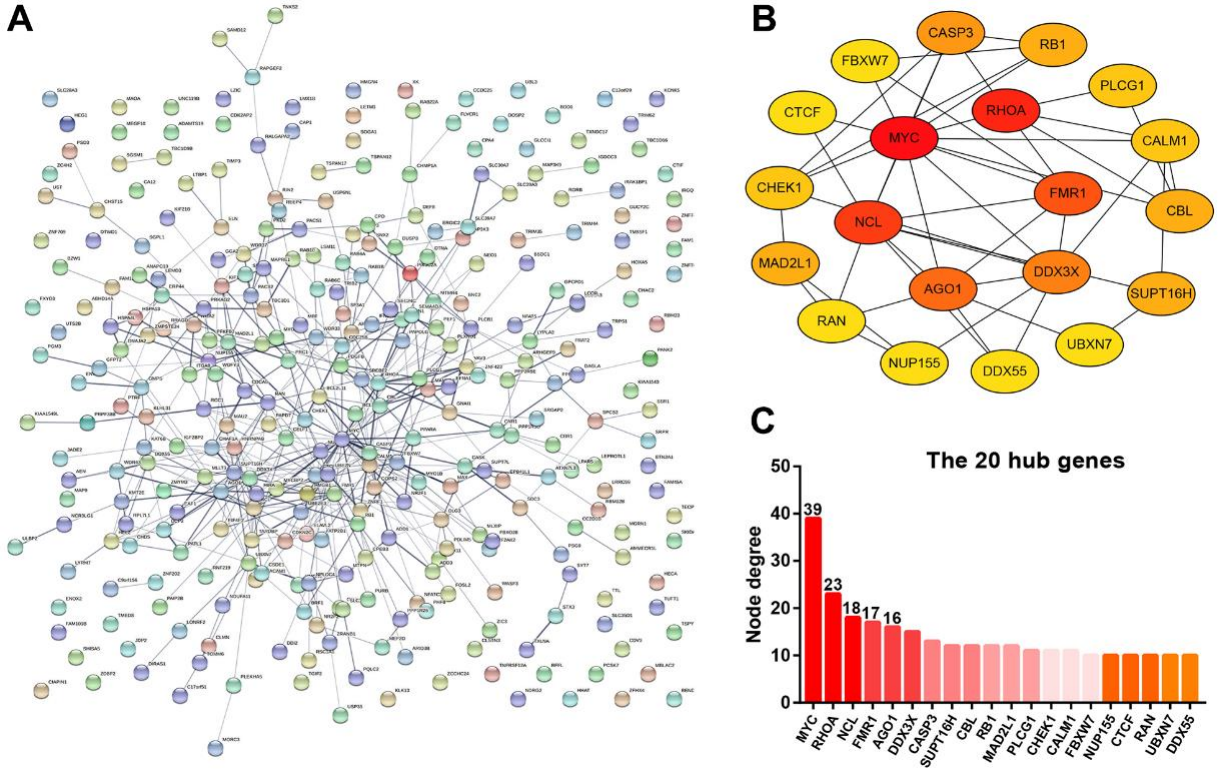
**Protein-protein interaction (PPI) analysis for the target genes of miR-5000-3p.** (**A**) The PPI network of target genes of miR-5000-3p established by STRING database. (**B**) The sub-PPI network of the top 20 hub genes among the target genes of miR-5000-3p constructed by Cytoscape. (**C**) The presentation of the top 20 hub genes and their corresponding node degree.

The target genes of miR-5000-3p were predicted by using a series of target gene prediction programs. Consequently, a total of 327 target genes were found to potentially bind to miR-5000-3p. To understand the molecular interaction of these target genes, STRING database was employed. As presented in Figure 6A, a PPI regulatory network of miR-5000-3p’s target genes was constructed. Next, the interaction gene pairs from STRING database were re-entered into Cytoscape software. Using CytoHubba, the top 20 hub genes among all the target genes of miR-5000-3p were identified by calculating node degree, and a sub-PPI network of the top 20 hub genes were re-established as shown in Figure 6B. As presented in Figure 6C, according to node degree, *MYC, RHOA, NCL, FMR1* and *AGO1* ranked as the top 5 hub genes, which might act in metastasis of ccRCC.

### Analysis for the top 20 hub genes in ccRCC

**Figure 7:**
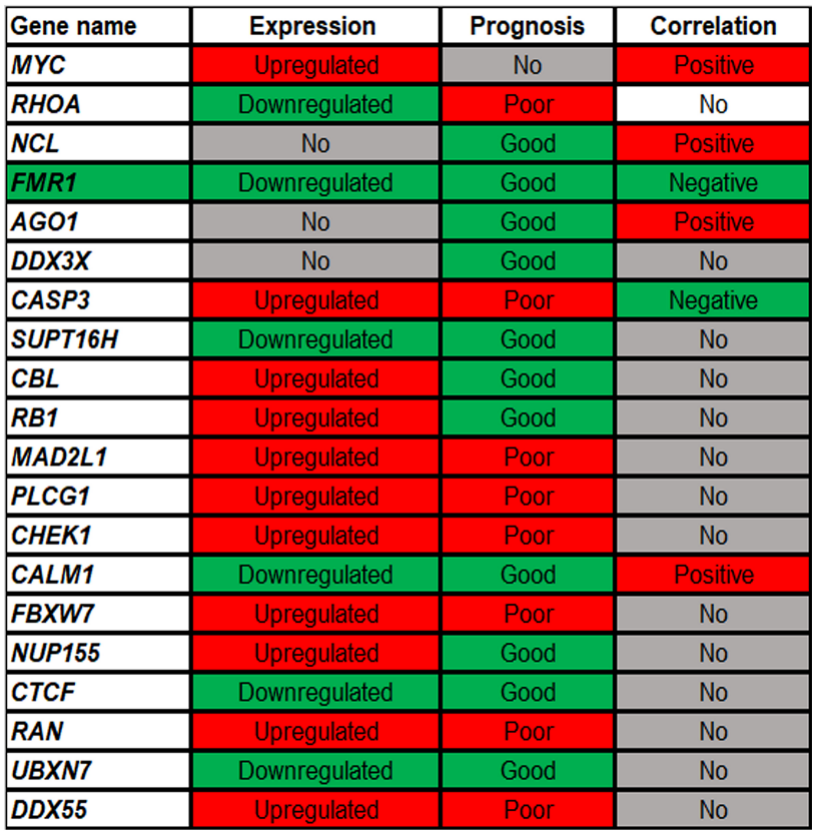
**The expression, prognosis and correlation landscape of the top 20 hub genes of the target genes of miR-5000-3p in ccRCC.** “Upregulated” and “Downregulated” mean that the expression of genes is significantly increased and decreased in ccRCC, respectively; “poor” and “good” represent the prognostic values of genes in ccRCC. “positive” and “negative” suggest the correlation relationship of gene-miRNA pairs in ccRCC.

Expression, survival and correlation analyses for the top 20 hub genes in ccRCC were successively performed (Figure 7). Expression analysis revealed that 11 genes (*MYC, CASP3, CBL, RB1, MAD2L1, PLCG1, CHEK1, FBXW7, NUP155, RAN* and *DDX55*) and 7 genes (*RHOA, FMR1, SUPT16H, CALM1, CTCF* and *UBXN7*) were significantly upregulated and downregulated in ccRCC when compared with normal controls, respectively. Survival analysis showed that 8 genes (*RHOA, CASP3, MAD2L1, PLCG1, CHEK1, FBXW7, RAN* and *DDX55*) and 11 genes (*NCL, FMR1, AGO1, DDX3X, SUPT16H, CBL, RB1, CALM1, NUP155, CTCF* and *UBXN7*) indicated poor and good prognosis in ccRCC patients, respectively. Correlation analysis demonstrated that only two genes (*FMR1* and *CASP3*) were significantly negatively associated with miR-5000-3p in ccRCC. Taken together, *FMR1* might be the most potential downstream gene of hsa_circ_0037858/miR-5000-3p axis related to metastasis of ccRCC.

### Establishment of a potential hsa_circ_0037858/miR-5000-3p/FMR1 axis related to metastasis of ccRCC

**Figure 8:**
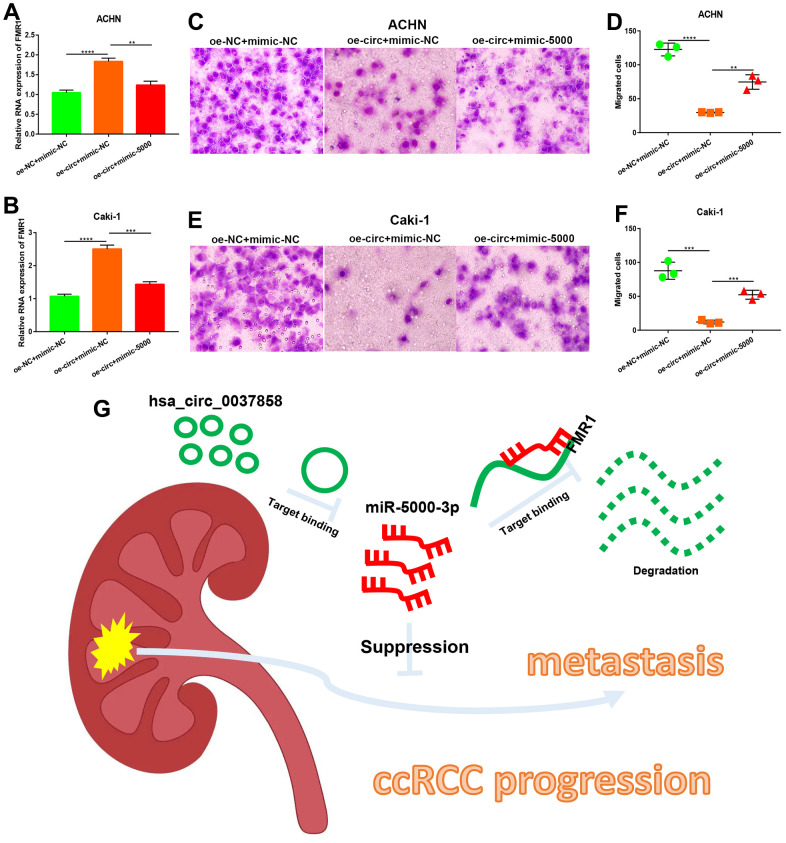
**Identification of a potential hsa_circ_0037858/miR-5000-3p/FMR1 axis contributing to metastasis of ccRCC.** The regulatory role of hsa_circ_0037858/miR-5000-5p in *FMR1* expression in ACHN (**A**) or Caki-1 (**B**). The inhibition of hsa_circ_0037858 in *in vitro* migration could be reversed by miR-5000-3p overexpression in ACHN (**C**, **D**) and Caki-1 (**E**, **F**) cell lines (**G**) The action model of hsa_circ_0037858/miR-5000-3p/*FMR1* axis in metastasis of ccRCC. **P<0.01; ***P<0.001; ****P<0.0001.

To preliminarily validate the identified circRNA/miRNA/mRNA axis, qRT-PCR assay was employed. As presented in Figure 8A, 8B, overexpression of hsa_circ_0037858 could significantly increase *FMR1* expression in both ACHN and Caki-1 cells. However, this upregulation could be markedly reversed after miR-5000-3p overexpression in ccRCC. Functional assay showed that increased expression of hsa_circ_0037858 significantly inhibited *in vitro* metastasis of ACHN and Caki-1 cells (Figure 8C–8F). Moreover, upregulation of miR-5000-3p could weaken hsa_circ_0037858-mediated inhibition of *in vitro* metastasis of ccRCC cells. Taken all these findings into consideration, hsa_circ_0037858/miR-5000-3p/*FMR1* axis might be a potential pathway involved in suppression of ccRCC metastasis as shown in Figure 8G.

## DISCUSSION

ccRCC is the most frequent histological subtype among all kidney malignancies, which is notorious for its high metastatic potential. Recently, numerous studies have reported the critical role of circRNA in cancer initiation and progression. However, the knowledge of circRNA in ccRCC metastasis is still inadequate and needs to be further explored.

At the beginning of this study, the circRNAs that might be associated with metastasis of ccRCC were screened out by combination of two GEO datasets, consisting of GSE100186 and GSE137836. After performing expression validation, hsa_circ_0037858 that was significantly downregulated in ccRCC was selected as the most potential circRNA involved in ccRCC metastasis. A previous study showed that hsa_circ_0037858 might function as a key regulator of progression of lumbar disc degeneration [27]. However, to date, its role in cancer, including ccRCC, has not been reported.

It has been widely acknowledged that miRNAs are involved in circRNA’s biological effects [28–30]. Intriguingly, CSCD database [21] analysis revealed that hsa_circ_0037858 possessed potential microRNA response elements. After performing binding miRNA prediction, miR-3064-5p, miR-6504-5p, miR-345-5p and miR-5000-3p were selected as candidate binding miRNAs of hsa_circ_0037858.

Based on competing endogenous RNA hypothesis [31], the binding miRNA of downregulated hsa_circ_0037858 should be upregulated in ccRCC. Consequently, among the 4 miRNAs, only miR-5000-3p expression was markedly increased in ccRCC compared with normal controls. Several studies have confirmed the oncogenic role of miR-5000-3p in human malignancies. For example, Zhuang et al. suggested that miR-5000-3p facilitated oxaliplatin resistance by targeting USP49 in colorectal cancer [32]; Chen et al. indicated that overexpression of miR-5000-3p enhanced proliferation and migration of laryngocarcinoma [33]. Furthermore, ROC curve analysis demonstrated that miR-5000-3p possessed significant diagnostic value in ccRCC.

Previous studies have showed that miRNAs exert their roles by negatively modulating target genes’ expression and function [34–36]. Therefore, the downstream target genes of miR-5000-3p were predicted using a series of target gene prediction programs. PPI network analysis showed close connection among these target genes, and *MYC, RHOA, NCL, FMR1* and *AGO1* were identified as the top 5 hub genes. Intriguingly, after performing expression, survival and correlation analysis, *FMR1* was considered as the most potential downstream target gene of hsa_circ_0037858/miR-5000-3p axis in ccRCC metastasis. To date, the function of *FMR1* in ccRCC has not been uncovered and needs to be further studied.

Our preliminary experimental validation showed that overexpression of miR-5000-3p could reverse hsa_circ_0037858-mediated *FMR1* upregulation in ccRCC cells. Moreover, after increased expression of miR-5000-3p, hsa_circ_0037858-induced suppression of *in vitro* metastasis of ccRCC cells was also markedly weakened. All these findings elucidated a potential role of hsa_circ_0037858/miR-5000-3p/*FMR1* axis in inhibition of ccRCC metastasis. However, there are still some limitations in the current work: (1) *in vivo* assays (including animal) were not conducted to confirm these findings; (2) clinical samples of metastatic ccRCC were not used to validate circRNA expression. Thus, our team and other group should further conduct much more basic experiments and large clinical trials to confirm the current findings in the future.
